# Quantitative analysis of mycosporine-like amino acids in marine algae by capillary electrophoresis with diode-array detection

**DOI:** 10.1016/j.jpba.2017.01.053

**Published:** 2017-02-04

**Authors:** Anja Hartmann, Adele Murauer, Markus Ganzera

**Affiliations:** Institute of Pharmacy, Pharmacognosy, University of Innsbruck, 6020 Innsbruck, Austria

**Keywords:** Algae, Sunscreen compounds, Mycosporine-like amino acids, Capillary electrophoresis

## Abstract

Marine species have evolved a variety of physical or chemical strategies to diminish damage from elevated environmental ultraviolet radiation. Mycosporine-like amino acids, a group of widely distributed small water soluble compounds, are biologically relevant because of their photo-protective potential. In addition, presumed antioxidant and skin protective strategies raise the interest for possible medicinal and cosmetic applications. In this study the first CE method for the quantification of mycosporine-like amino acids in marine species is presented. A borate buffer system consisting of 30 mM sodium tetraborate in water at a pH-value of 10.3 enabled the baseline separation of five MAAs, namely palythine, mycosporine-serinol, asterina-330, shinorine and porphyra-334, in 27 min. Separation voltage, temperature and detection wavelength were 25 kV, 25 °C and 320 nm, respectively. The optimized method was fully validated and applied for the quantitative determination of MAAs in the marine macroalgae *Palmaria palmata*, *Porphyra umbilicalis*, and *Porphyra sp*., as well as the lichen *Lichina pygmaea*.

## 1 Introduction

The stratospheric ozone layer offers substantial protection to all living organisms by absorbing high doses of harmful solar ultraviolet radiation. This effective shield has been depleted for decades due to the use of so-called greenhouse gases, various industrial halogenated chemicals like chlorofluorocarbons that unintentionally reacted with ozone [1,2]. As a result high levels of harmful solar radiation in particular UV-B (280–315 nm) and UV-A (315–400 nm) are reaching the earth’s surface, with severe consequences for humans (accelerated skin ageing, increasing numbers of skin cancer, etc.) as well as the environment [1–3]. Protection strategies to counteract the damaging effects of ultraviolet radiation in living organisms are diverse ranging from repair mechanisms to the synthesis of cellular metabolites [4–6]. In microorganisms, mycosporine-like amino acids (MAAs) and their precursors represent one of the most relevant compounds in this respect [7,8]. They have predominantly been isolated from algae and cyanobacteria, and due to their high molar absorption coefficients up to 50,000 M^−1^ cm^−1^ as well as antioxidative properties they serve as microbial sunscreens [9,10]. Recent in vitro data also indicated skin protective and wound healing effects of MAAs [11–13]; for example porhyra-334 was able to suppress ROS (reactive oxygen species) production in human skin fibroblast cells [14]. Therefore, the quantitative determination of MAAs in algae is an ecologically and pharmaceutically interesting task. A few already published RP-HPLC methods face the problem that MAAs are highly polar and thus barely retained on the stationary phase [15]. Just recently the first validated HILIC (hydrophilic interaction chromatography) method has been presented [16]. The option of using capillary electrophoresis (CE) has never been considered, even if MAAs seem to be ideal targets due to their ionizable nature just like amino acids [17–19]. Accordingly, in this study the overall first separation of MAAs by CE is presented.

## 2 Experimental

### 2.1 Reagents, standards and samples

All solvents/chemicals used for the isolation of MAAs (see [12] for details on sources and purification protocols, and [16] for NMR data), as well as CE method development and sample preparation were of analytical grade and purchased from Merck (Darmstadt, Germany). HPLC grade water was produced by a Sartorius arium 611 UV water purification system (Göttingen, Germany). Three of the algae investigated, namely *Palmaria palmata* (Irish Seaweeds, Belfast, UK), *Porphyra sp.* (Asia Express Food, Kampen, NL) and *Porphyra umbilicalis* (ALGAS Atlanticas Algamar SL, Poligono De Amoedo, Spain) were commercially available. *Leptolyngbya foveolarum* was obtained as pure strain from CCALA – Culture Collection of Autotrophic Organisms, Institute of Botany, Academy of Sciences, Trebon, Czech Republic (Strain-Nr: 132). One sample of a marine lichen (*Lichina pygmea*) was kindly provided by Professor Ulf Karsten, University of Rostock, Germany.

### 2.2 Sample preparation

Dried algal material was crushed to powder in a grinding mill and sieved prior to extraction to guarantee homogeneity. The samples (about 75 mg) were subsequently extracted for 30 min with 5 mL methanol/water (25:75) in a falcon tube by sonication (Bandelin Sonorex 35 KHz, Berlin, Germany). After centrifugation at 1400 × *g* for 10 min the supernatant was collected. This extraction procedure was repeated twice and the combined solutions were evaporated to dryness at 45 °C in a vacuum evaporator (Büchi, Flawil, Switzerland). Prior to CE analysis the extract was dissolved in 5.00 mL HPLC grade water. All sample solutions were membrane filtered (0.45 µm, Minisart, Sartorius, Göttingen, Germany) and each standard or sample solution was analyzed in triplicate. All solutions were stable for at least for 1 week if stored at 4 °C (confirmed by re-assaying).

### 2.3 Analytical conditions

Analytical experiments were performed on a 3D-CE system from Agilent (Waldbronn, Germany), equipped with autosampler, diode array detector (DAD) and temperature controlled column compartment. Separations were conducted in fused-silica capillaries (75 µm i.d.; 80 cm effective length, 85.5 cm total length) purchased from PolymicroTechnologies (Phoenix, AZ, USA). For optimum results a 30 mM sodium tetraborate decahydrate (borax) solution with a pH of 10.3 (adjusted with 1 M NaOH solution) was used. All samples were injected in hydrodynamic mode (50 mbar for 4 s), and separation voltage, temperature and detection wavelength were set to 25 kV, 25 °C, and 320 nm. The required run time was 30 min; between runs the capillary was flushed with 0.01 M NaOH solution, water and buffer for 3 min each. After the analysis of approx. 15 samples a longer washing step (15 min per solvent) was required to ensure a stable current and reproducible results. New capillaries were rinsed with 0.1 M NaOH and water (30 min each) prior to initial use. Before CE analysis all samples, buffers and washing solutions were membrane filtered.

### 2.4 Method validation

The optimized CE-method was validated according to ICH guidelines [20]. To obtain calibration curves three reference compounds, which were available in sufficient amount, were dissolved in water and individual concentration levels prepared by serial dilution with the same solvent. Limit of detection (LOD) and limit of quantification (LOQ) were determined corresponding to concentrations equivalent to S/N ratios of 3 (LOD) and 10 (LOQ). Selectivity was assured by utilizing the peak-purity option provided in the operating software (Agilent chemstation version RevB.04.04-Sp2). Repeatability was determined by the relative standard deviation from several measurements of the same solution, and intermediate precision was investigated by analysing five individually prepared samples of *Porphyra sp.* on one day. The same procedure was carried out on two consecutive days in order to calculate the inter-day precision. Accuracy was assured by spiking one sample with known amounts of standard compounds at three concentration levels prior to extraction. Table 1 summarizes all validation data.

## 3 Results and discussion

### 3.1 Method development and optimization

**Fig. 1:**
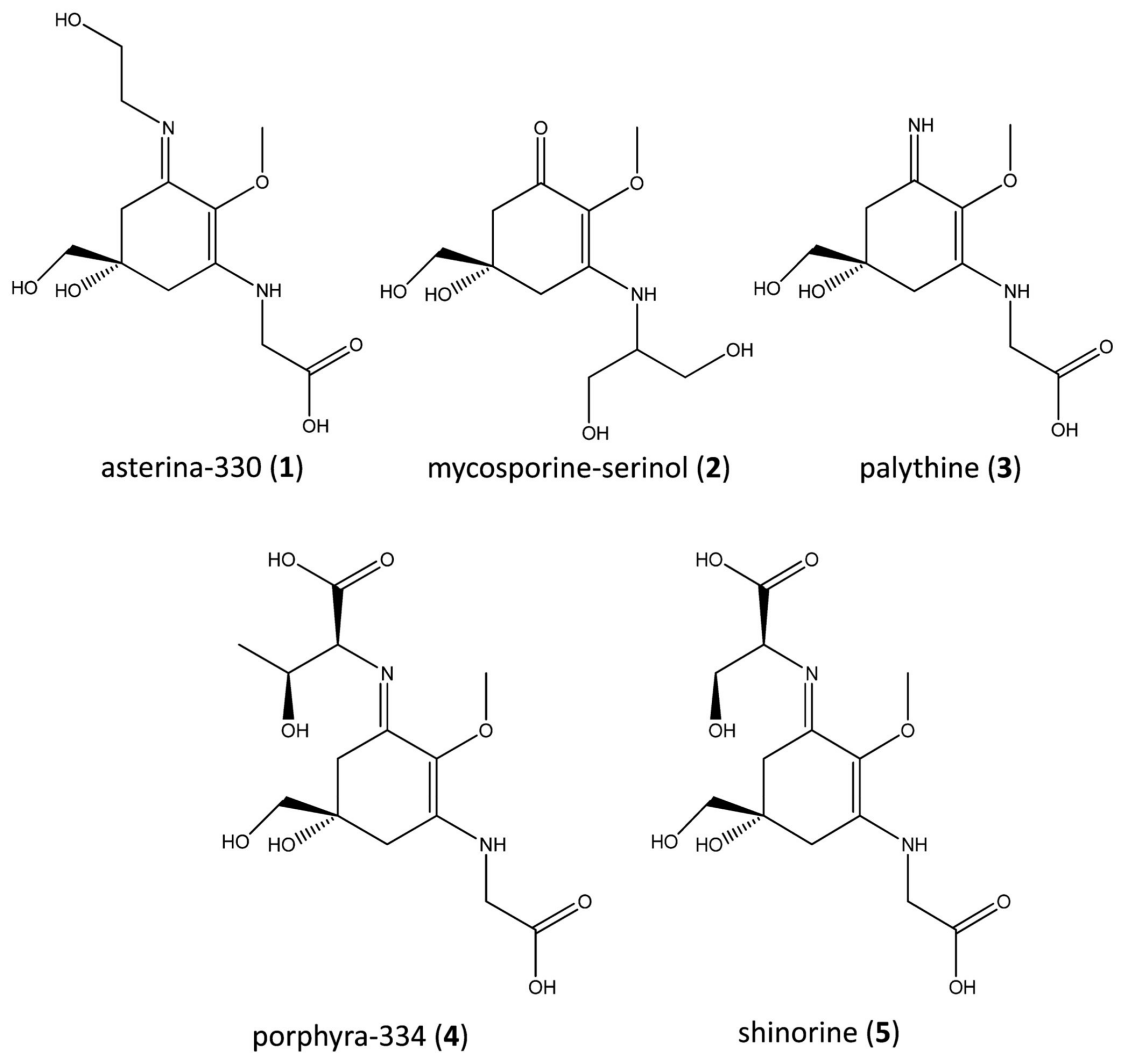
Structures of the investigated MAAs, numbered in their CE migration order.

**Fig. 2:**
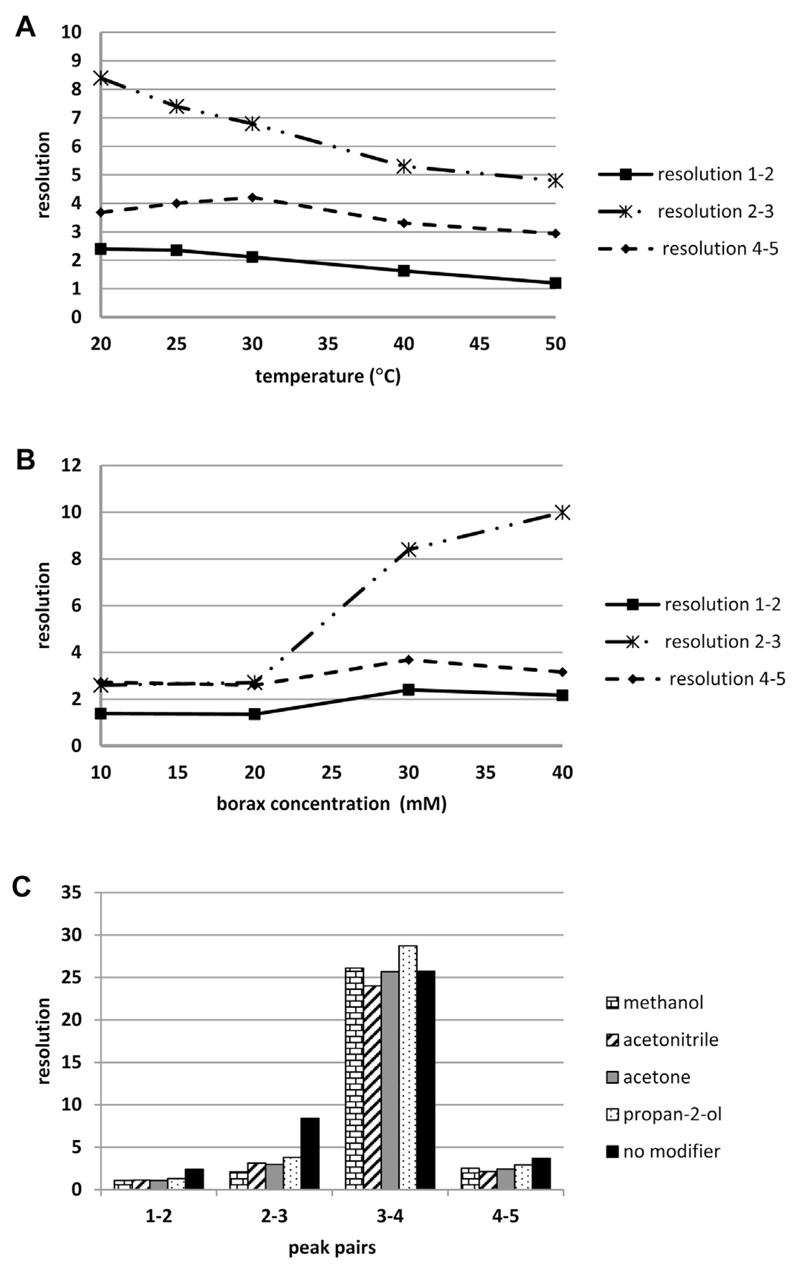
Influence of temperature (A), buffer molarity (B) and modifiers (C) on the resolution (*R*_s_) of relevant signals. Values represent the mean of three injections.

Based on the fact that no literature was available for the separation of MAAs by capillary electrophoresis several electrolyte systems were initially tested including acetate (pH 4.0–5.5), phosphate (pH 5.5–7.5), Tris (pH 7.5–9.5) and borate buffers (pH 8.5–11.0). Respective experiments were performed using a standard mixture of five in-house isolated standard compounds, namely asterina-330 (**1**), mycosporine-serinol (**2**), palythine (**3**), porphyra-334 (**4**) and shinorine (**5**); see Fig. 1 for structures. Only with an aqueous borate buffer system at a pH higher than 10 they could be resolved; 10.3 was selected because peak separation was found to be optimal. The impact of other separation parameters like temperature, buffer molarity or the effect of modifiers is shown in Fig. 2; peak-pair **3**–**4** is sometimes not depicted as its *R*_s_-value (resolution) was always higher than 10. Concerning the influence of temperature it was observed that resolution generally increased when lower temperatures were applied (Fig. 2A). For example the separation of the neighbouring peak pair porphyra-334 (**4**) and shinorine (**5**) was significantly better at 30 °C (*R*_s_ = 4.21) compared to 50 °C (*R*_s_ = 2.94). Below 30 °C the most critical peak pair **1**–**2** was slightly better resolved; with *R*_s_ values ≥2.35 for all MAAs 25 °C was considered to be the optimum, because at lower temperatures an increasingly noisy baseline was observed. The impact of buffer molarity was investigated from 10 to 40 mM (Fig. 2B). By trend the resolution between individual MAAs decreased with increasing molarity. This can be explained by a steadily decreasing electroosmotic flow (EOF), which prolongs migration times and thus results in broader peaks. With a 30 mM borate buffer all standards, including compounds **1** and **2**, were baseline resolved in less than 28 min. Molarities below this value were less favourable as asterina-330 and mycosporine-serinol, which show very similar migration times, started to overlap. Additionally, the influence of modifiers on the resolution was investigated (Fig. 2C). For this purpose different solvents like methanol, acetone or acetonitrile were added in a concentration of 10% to the optimal borax buffer; however, none of them generally or significantly improved the results. In order to enable fast separations a voltage of 25 kV was applied and 320 nm was selected as all target analytes could be detected sensitively (see supporting information for UV–vis spectra of MAAs).

### 3.2 Method validation and quantification of MAAs

Three of the five MAAs were available in larger amount and thus could be used for method validation. By accurately weighing 1.0 mg of palythine (**3**), porphyra-334 (**4**) and shinorine (**5**) and dissolving them in 1.0 mL water a stock solution was prepared. Individual calibration levels were obtained by its serial dilution, and each of them was analyzed under optimum CE conditions in triplicate. In a concentration range of approx. 1000 to 15 µg/mL the coefficient of determination (*R*^2^) was always higher than 0.990 (Table 1). LOD and LOQ, which were determined by comparing measured signals from standard solutions with known low concentrations with those of blank samples, were found to be below 4.8 and 14.6 µg/mL, respectively. Accuracy was determined by spiking individual *L. foveolarum* samples with known amounts of the reference compounds at three concentration levels (500, 250 and 31.25 µg/mL). For all compounds and spike levels except the highest concentrations of porphyra-334 and shinorine the determined recovery rates were in the range of ±5% of the theoretical value. Repeatability of the assay was concluded by relative standard deviations below 4.64% when analysing the same sample solutions, intermediate precision was investigated inter-day and intra-day. The latter revealed that both were in an acceptable range for the three MAAs present in *Porphyra sp*.; e.g. for porphyra-334 the intra-day variance was ≤6.35%, inter-day precision was 4.22%. Finally, the methods selectivity was confirmed by two facts. All quantified compounds appeared as symmetrical signals without shoulders, and secondly no indications for co-migration were found by evaluating the respective UV-spectra using the peak purity option in the software.

**Fig. 3:**
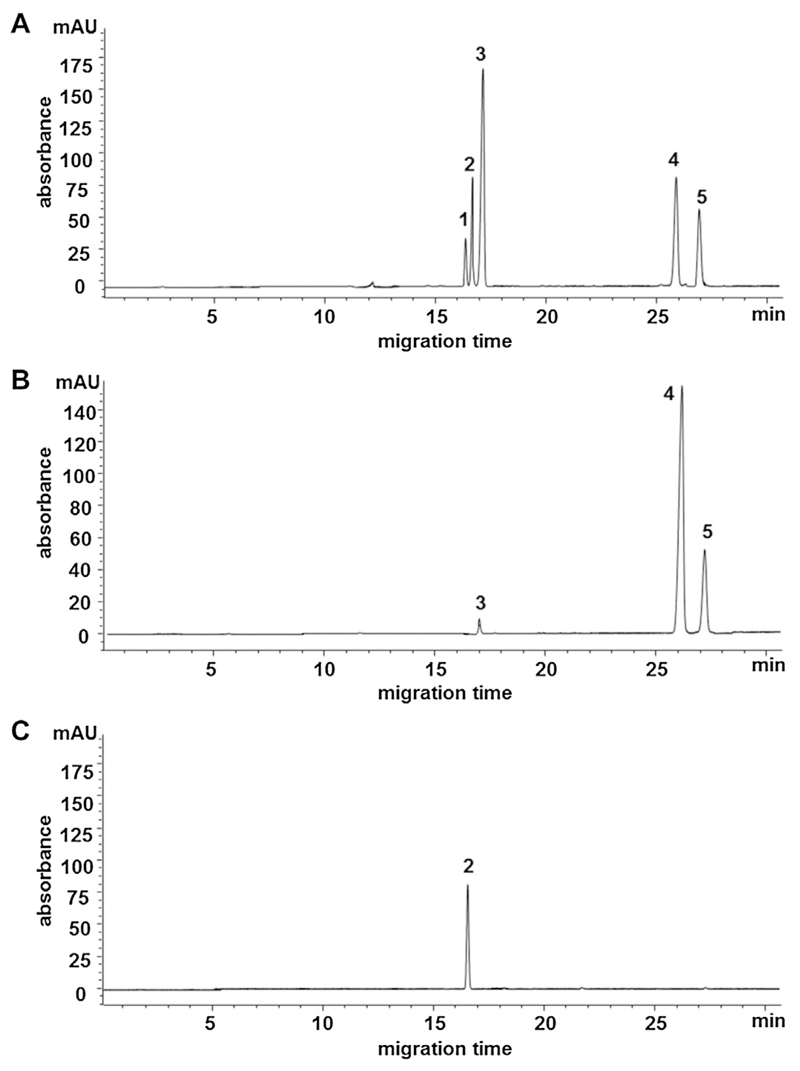
CE-separation of a standard mixture containing 5 MAAs (A) and two sample solutions (B: *Porphyra sp*. and C: *Lichina pygmeae*) under optimized conditions (capillary: fused-silica, 85.5 cm × 75 μm I.D.; buffer: 30 mM borax, pH 10.3; hydro-dynamic injection at 50 mbar for 4 s; separation voltage: 25 kV; temperature: 25°C; detection wavelength: 320 nm). Assignment of signals according to Fig. 1.

**Fig. 4:**
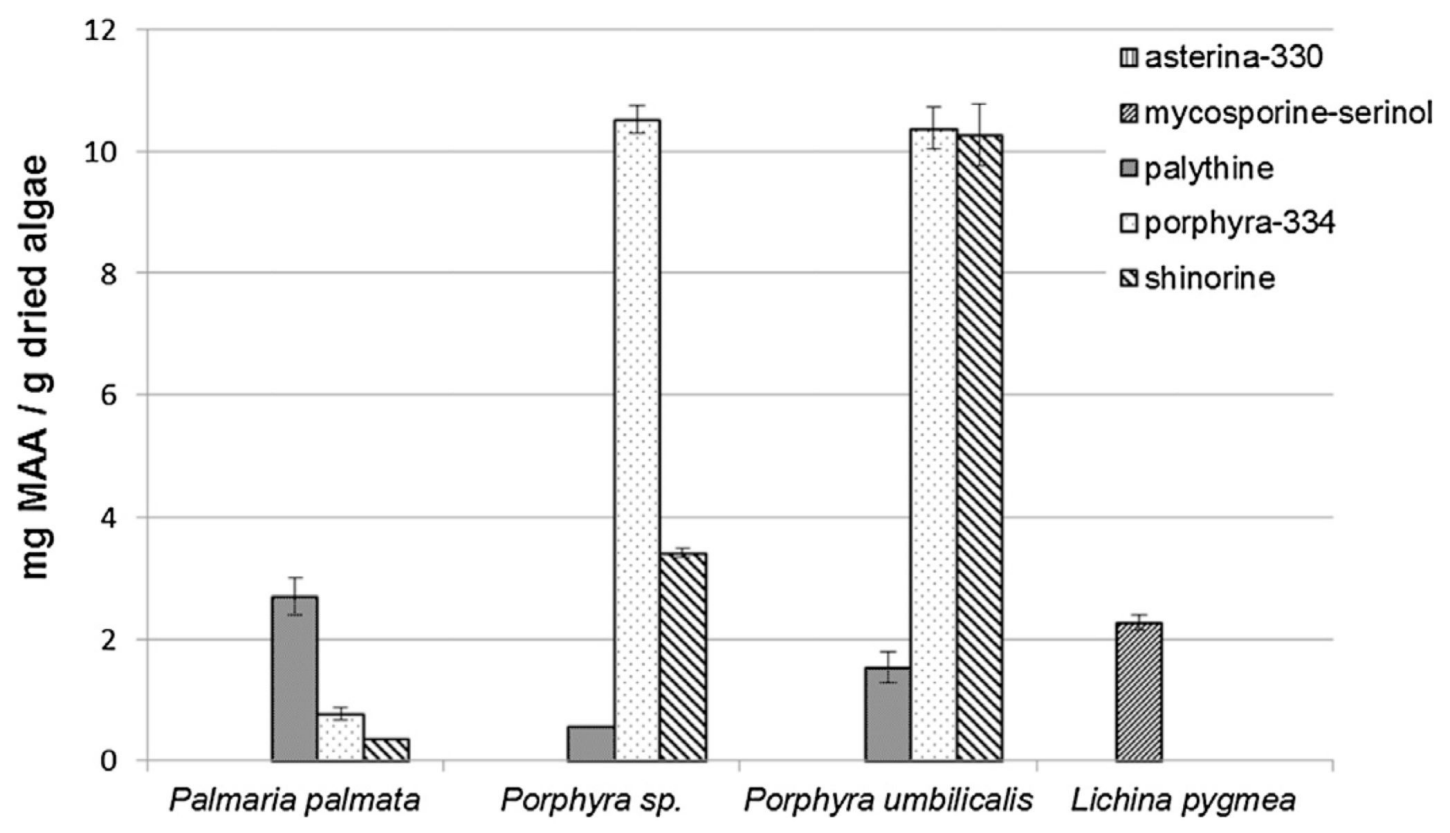
Quantification of MAAs in different samples. Results are stated as mg MAA/g dry weight of algae, and the standard deviation is indicated by bars (*n* = 3).

The application of the developed CE assay on real samples is shown in Fig. 3, and quantitative results are presented in Fig. 4. In this respect it has to be noted that mycosporine-serinol (**2**) in *Lichina pygmaea* was quantified using the calibration data of porphyra-334 (**4**). Besides this lichen several marine red macroalgae were analyzed for their MAA content. For this purpose commercially available species of the *Porphyra* genera and *Palmaria palmata* were chosen because of their relevance as food items; e.g. *P. palmata* is known in Ireland as “dulse”, *Porphyra* is used to prepare “nori”. The same batch of *Porphyra sp*. has been analyzed for its MAA content in a previously published HPLC study [15] and the quantitative results for the three major MAAs porphyra-334 (CE: 10.53 mg/g, HPLC: 10.85 mg/g), shinorine (CE: 3.41 mg/g, HPLC: 3.27 mg/g) and palythine (CE: 0.56 mg/g, HPLC: 0.48 mg/g) are well comparable. In *Palmaria palmata* the major MAA is palythine with a concentration of 2.71 mg/g dried algae. The content of mycosporine-serinol in *Lichina pygmaea* was determined to be 2.27 mg/g; none of the samples contained quantifiable amounts of asterina-330.

## 4 Conclusions

In this article the first CE-method for MAA analysis is presented. All determined validation parameters were within acceptable limits and the quantitative results were accurate, reproducible and in agreement to published data. Compared to the common HPLC protocols the CE assay has distinct advantages. On conventional RP phases MAAs are hardly retained and therefore co-elutions not unlikely. Using HILIC as an alternative has been described [16], yet this approach requires long column equilibration and the peaks are comparatively broad. These points are not relevant for CE, which additionally is extremely economic in operation (e.g. only a few nano litre of sample solution are required for analysis). However, this is also the reason for generally higher detection limits in CE especially if used together with a DAD; CE-MS could not be utilized in the current case as the optimal buffer is nonvolatile. In summary, the option of using CE for the analysis of MAAs is an interesting one, which definitely will help to expand the knowledge on this biologically and pharmacologically highly interesting group of natural products.

## Supplementary data

The UV–vis spectra of MAAs analyzed in this study are shown as supplementary information.

**Appendix A. Supplementary data**

Supplementary data associated with this article can be found, in the online version, at doi:10.1016/j.jpba.2017.01.053.

supplementary

## Figures and Tables

**Table 1:** Validation results for three MAAs.

Parameter/compound	Palythine	Porphyra-334	Shinorine
Regression equation	*Y* = 1.607*x* − 20.876	*Y* = 3.625*x* − 96.562	*Y* = 3.184*x* − 57.236
*σ*_rel_ of slope	1.36	4.36	2.88
*R*^2^	0.995	0.994	0.995
Range (µg/mL)	950–14.8	1060–16.6	1100–17.2
LOD (µg/mL)	4.3	4.4	4.8
LOQ (µg/mL)	12.9	13.3	14.6
Accuracy^a^			
High spike	99.26	106.36	105.53
Medium spike	101.46	97.11	102.06
Low spike	96.81	98.55	101.83
Precision^b^			
Intra-day	3.94	6.35	5.87
Inter-day	2.23	4.22	4.85

Abbreviations: *Y* = peak area, *x* = concentration (µg/mL), *σ*_rel_ = relative standard deviation, *R*^2^ = determination coefficient, LOD = limit of detection, LOQ = limit of quantification.

a Expressed as recovery rates in percent (sample: *Leptolyngbya foveolarum*).

b Maximum relative standard deviation (peak area) within one and three consecutive days (*n* = 5; sample: *Porphyra sp.*).

## Abbreviations

CE: capillary electrophoresis
DAD: diode array detector
EOF: electroosmotic flow
ICH: International Council for Harmonisation of Technical Requirements for Pharmaceuticals for Human Use
HILIC: hydrophilic interaction chromatography
LOD: limit of detection
LOQ: limit of quantification
MAA: mycosporine-like amino acids
ROS: reactive oxygen species
S/N: signal-to-noise
*R*^2^: coefficient of determination
*R*_s_: resolution
Tris: tris(hydroxymethyl)aminomethane
