# Supplementary material for: Quantitative analysis of mycosporine-like amino acids in marine algae by capillary electrophoresis with diode-array detection
Source: J Pharm Biomed Anal. Author manuscript; Available in PMC 2017 May 10. (PMC5388179; doi:10.1016/j.jpba.2017.01.053)

Quantitative analysis of mycosporine-like amino acids in marine algae by capillary electrophoresis with diode-array detection

**Supplementary information**

Anja Hartmann, Adele Murauer, Markus Ganzera^*^

**Affiliation**

Institute of Pharmacy, Pharmacognosy, University of Innsbruck, 6020 Innsbruck, Austria

*Corresponding author:

Assoc. Prof. Dr. Markus Ganzera

Institute of Pharmacy, Pharmacognosy, University of Innsbruck

Innrain 80-82, 6020 Innsbruck, Austria

Phone: 0043-512-507 58406 (fax: 58499), E-mail: [markus.ganzera@uibk.ac.at](mailto:markus.ganzera@uibk.ac.at)

**Appendix A.**

The table summarizes the UV-spectra and absorption maxima of MAAs analysed in this study.


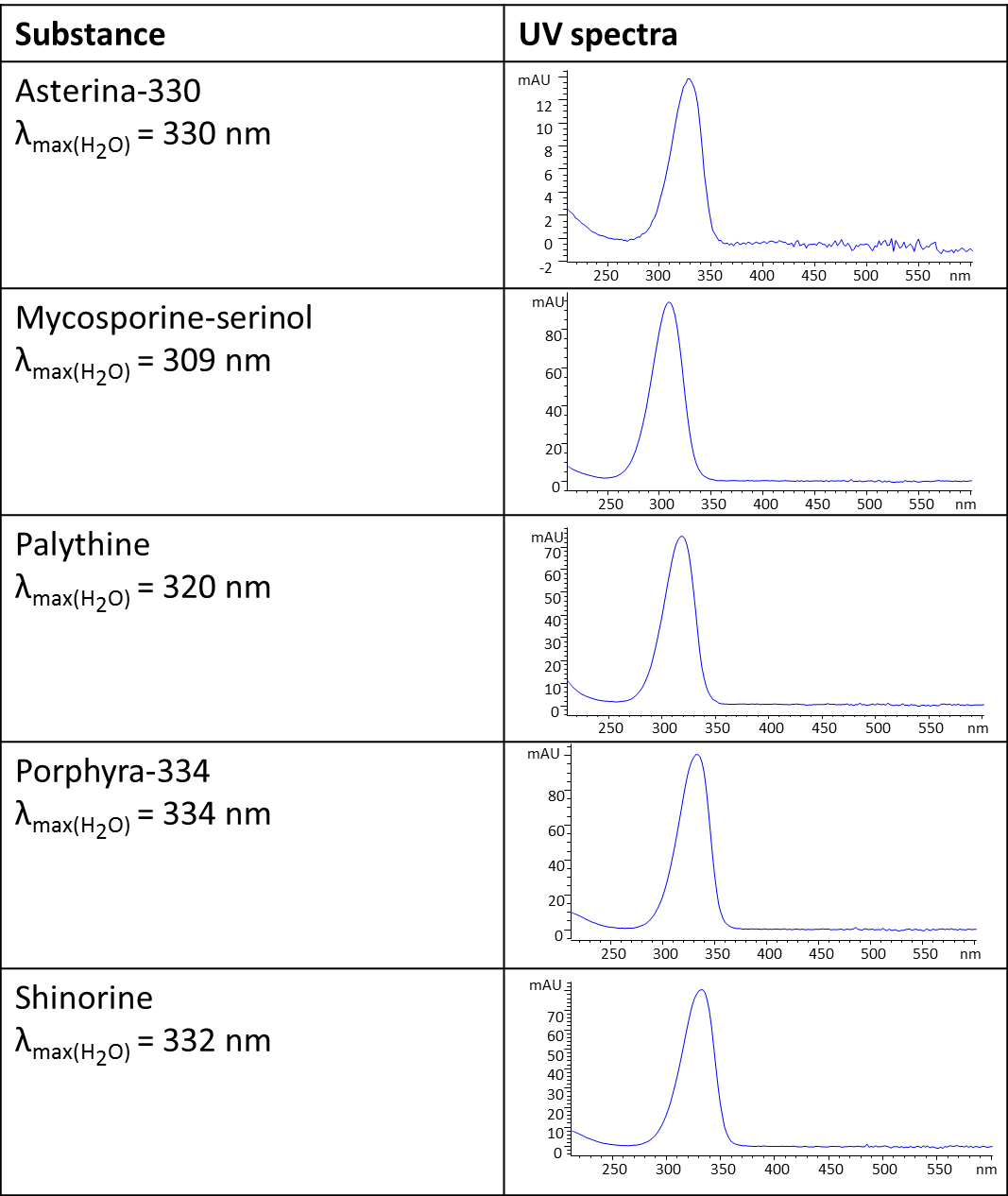

Supplement: supplementary [file NIHMS72232-supplement-supplementary.docx]
